# COMPARING THE EFFECTS OF BIOFEEDBACK AND TRANSCUTANEOUS TIBIAL NERVE STIMULATION, ALONE OR IN COMBINATION, IN WOMEN WITH COEXISTING COMPLAINTS OF DYSSYNERGIC DEFECATION AND STRESS URINARY INCONTINENCE: A RANDOMIZED CONTROLLED TRIAL

**DOI:** 10.1590/S0004-2803.24612025-123

**Published:** 2026-08-17

**Authors:** Neda ORAKIFAR, Fatemeh ORAKI KOOHSHOORI, Nahid PIRAYEH, Eskandar HAJIANI, Shahla ZAHEDNEJAD

**Affiliations:** 1 Ahvaz Jundishapur University of Medical Sciences, Rehabilitation Research Center, Ahvaz, Iran.; 2 Ahvaz Jundishapur University of Medical Sciences, School of Rehabilitation Sciences, Department of Physiotherapy, Ahvaz, Iran.; 3 Ahvaz Jundishapur University of Medical Sciences, Clinical Sciences Research Institute, Alimentary Tract Research Center, Ahvaz, Iran.

**Keywords:** Dyssynergic defecation, constipation, stress urinary incontinence, biofeedback, transcutaneous tibial nerve stimulation, Defecação dissinérgica, constipação, incontinência urinária de esforço, biofeedback, estimulação transcutânea do nervo tibial

## Abstract

**Objective:**

To compare the efficacy of electromyography biofeedback (EMG-BF), transcutaneous tibial nerve stimulation (TTNS), and combination of EMG-BF with TTNS in women with stress urinary incontinence (SUI) and dyssynergic defecation (DD) disorders.

**Methods:**

In this single-blinded, three-arm, randomized controlled trial, eighty-seven women (18-60 years) with concurrent SUI and DD disorders randomly allocated into three groups (29 per group) including TTNS, EMG-BF and combined treatment group. Participants in each group received fifteen treatment sessions (3 day per week with at least 48-hour break between sessions). Wexner constipation scoring system questionnaire, 3-day bladder diary and EMG activity level of the pelvic floor muscle (PFM) during maximal voluntary contraction (MVC), straining, and coughing were assessed as treatment outcomes measures at baseline and immediately after sessions 5,10 and 15.

**Results:**

A two-way repeated measure analysis of variance showed that group interaction by time was significant for all measured variables. Further analysis by the Tukey post hoc test showed significant difference of Wexner questionnaire, 3-day bladder diary and EMG activity level values in the EMG-BF group compared with TTNS group (*P*<0.001) and in combined treatment group compared with TTNS group (*P*<0.001) in sessions 10 and 15.

**Conclusion:**

EMG-BF and combined treatment significantly improved defecatory and urinary related symptoms and PFM performance in women with SUI and DD disorders.

## HIGHLIGHTS

• EMG-BF, either alone or in combination with TTNS, significantly improved defecatory and urinary symptoms.

• EMG-BF, either alone or in combination with TTNS, significantly improve pelvic floor muscle activity.

• A minimum of ten treatment sessions is required to achieve clinically meaningful improvements.

## RESUMO

**Objetivo:**

Comparar a eficácia do biofeedback eletromiográfico (EMG-BF), da estimulação transcutânea do nervo tibial (TTNS) e da combinação de EMG-BF com TTNS em mulheres com incontinência urinária de esforço (IUE) e distúrbios de defecação dissinérgica (DD).

**Métodos:**

Neste ensaio clínico randomizado, controlado, de três braços e simples-cego, oitenta e sete mulheres (18-60 anos) com IUE e DD concomitantes foram randomicamente alocadas em três grupos (29 por grupo): TTNS, EMG-BF e tratamento combinado. As participantes de cada grupo receberam quinze sessões de tratamento (3 dias por semana, com intervalo mínimo de 48 horas entre as sessões). O questionário do sistema de pontuação de constipação de Wexner, o diário miccional de 3 dias e o nível de atividade eletromiográfica (EMG) dos músculos do assoalho pélvico (MAP) durante contração voluntária máxima (CVM), esforço evacuatório e tosse foram avaliados como medidas de desfecho no início do estudo e imediatamente após as sessões 5, 10 e 15.

**Resultados:**

A análise de variância de medidas repetidas em dois fatores mostrou que a interação grupo versus tempo foi significativa para todas as variáveis analisadas. Análises adicionais pelo teste post hoc de Tukey demonstraram diferença significativa nos valores do questionário de Wexner, do diário miccional de 3 dias e dos níveis de atividade EMG no grupo EMG-BF em comparação ao grupo TTNS (P<0,001), bem como no grupo de tratamento combinado em comparação ao grupo TTNS (P<0,001) nas sessões 10 e 15.

**Conclusão:**

O EMG-BF e o tratamento combinado melhoraram significativamente os sintomas relacionados à evacuação e urinários, bem como o desempenho dos músculos do assoalho pélvico em mulheres com IUE e DD.

## INTRODUCTION

Urinary incontinence (UI), characterized by involuntary leakage of urine, is one of the most common clinical conditions related to pelvic floor dysfunction^1^. Even though it can be found in both genders, it has twice as much prevalent in women in comparison with men^2^. So, it affects between 25% and 45% of adult women, and has a profound impact on quality of life (QOL)^3^. When UI is associated with exertions that increase intraabdominal pressure, such as coughing, sneezing, squatting, weight lifting or laughing, the dysfunction is referred to as ‘stress urinary incontinence’ (SUI). In this condition, sufficient pelvic floor muscle (PFM) activity is impaired due to lack of ability to keep the bladder elevated during increased intraabdominal pressure and to maintain adequate urethral closure pressure preventing the urine leakage^4^. Indeed, PFM deficiency is one of the main causes of SUI^5^. Therefore, conservative methods that increase PFM performance (i.e., strength and endurance) and correct coordinated activity of the muscles have been recognized as the first-line treatment for SUI^6^. Among these, electromyography biofeedback (EMG-BF) therapy is a widely used conservative method, which has an important role in understanding the PFM position, facilitating the active muscle contractions, refining the PFM function^5^ ^,^ ^7^, as well as reducing the symptoms of SUI and consequently, improving the QOL^8^ ^,^ ^9^ ^,^ ^10^. Transcutaneous tibial nerve stimulation (TTNS) is another conservative modality used for treating the patients with UI. Given that the PFM and bladder sphincter are supplied by the same as nerve roots as posterior tibial nerve^11^, neuromodulating signals from the posterior tibial nerve can improve urinary function in patients^12^.

It is worth to mention that, the efficacy of these treatment modalities was often investigated in patients with complaint of UI symptoms. However, there is a high incidence of pelvic floor dysfunction symptoms coexistence^13^. Specifically, defecatory disorders are frequent among patients with UI, particularly in women^14^ ^,^ ^15^ ^,^ ^16^. The association between constipation and UI confirmed in a meta-analysis study^17^ and recommend that the constipation screening should be performed during the evaluation and management of women with urinary dysfunction^18^. About one-third of chronically constipated patients have an evacuation disorder and Dyssynergic defecation (DD) is the most common cause of the evacuation disorder^19^. Specifically, the inability to coordinate the contractions of anorectal muscles due to weak rectoabdominal propulsive force and failure to relax the puborectalis and external anal sphincter muscles lead to difficult and ineffective stool evacuation in patients with DD^20^ ^,^ ^21^. This condition can damage the PFM^22^ ^,^ ^23^, stretch the pelvic nerves, and move the anus and its surrounding structures downward^14^. Furthermore, the presence of the related forms and structures^24^, the neural overlap^25^, as well as the anatomical proximity of the urinary and intestinal tracts are the other reasons that support coexistence of the symptoms of defecatory and UI disorders^23^ ^,^ ^26^. Therefore, the provision of appropriate treatments for improving constipation symptoms has been emphasized when treating women with UI^17^. Recent systematic reviews displayed the positive effects of EMG-BF and TTNS in patients with chronic constipation^27^ ^,^ ^28^. However, no study has yet investigated the treatment effects of these modalities in patients with coexistence complaints of SUI and DD. The aim of current study was to compare efficacy of EMG-BF, TTNS, and combined treatment of EMG-BF with TTNS on constipation and urinary symptoms and EMG activity level of PFM in women with concurrent SUI and DD disorders.

## METHODS

### Study design

This study was conducted as an assessor-blinded, three-arm, randomized controlled trial in the Rehabilitation Research Center of Ahvaz Jundishapur University of Medical Science. This trial received ethical approval from the university’s institutional review board (IR.AJUMS.REC.1402.006) and was registered at https://irct.behdasht.gov.ir/ (reference number: IRCT20230228057560N1) before the recruitment of the first participant.

### Participant recruitment

**FIGURE 1:**
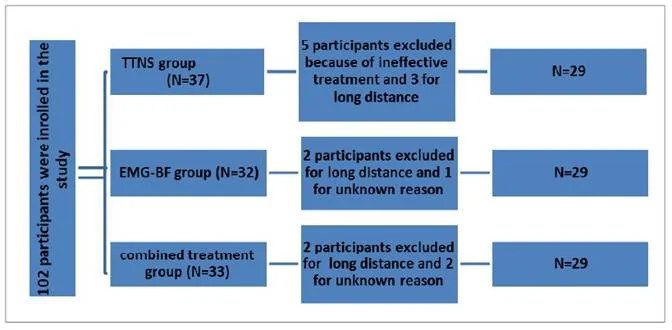
Participants’ flow diagram.

The sample size calculated based on previous related studies^29^ and the sample size determined as 23 subjects per each group to provide a power of 90% and a significance level of 5% for detecting the difference between three groups. A final sample size was set at 87 (29 each group), taking into consideration drop-outs. Participants were selected voluntarily and consecutively from outpatients visiting gynecologists and gastroenterologists’ offices whom organic and metabolic causes leading to chronic constipation were excluded by colonoscopy and laboratory examinations and by their diagnosis between May 2023 and August 2024. Women with complaints of SUI and DD symptoms from 6 months and more, who had failed to respond sufficiently to drug therapy and dietary or lifestyle changes were considered eligible to participate in the study if they (i) were not pregnant; (ii) were aged 18 to 60 years; (iii) were diagnosed with SUI by an urogynecologist and leakage episode recorded more than once a week; (iv) were diagnosed with DD on the basis of fulfilled the Rome III criteria for functional constipation and had complaining of decreased bowel frequency (fewer than three defecations per week), sensation of incomplete evacuation, lumpy or hard stools at least 25% of defecations, and straining during at least 25% of defecations. Women were excluded if they were (i) diagnosed with severe pelvic organ prolapse; (ii) within the 6-month postpartum period; (iii) experiencing urine retention or constipation as a medication side-effect; (iv) experiencing mixed or urge UI; (v) presenting with urinary tract infections and detrusor over activity; (vi) experiencing constipation secondary to obstructing neoplasm of the colon, hypothyroidism and anatomic or systemic disease; (vii) having the history of diabetes, cognitive problems, active bowel disease and fissure, severe primary diseases of cardiovascular, cerebrovascular, liver, kidney, and hematopoietic system and neurologic diseases such as multiple sclerosis, stroke, or spinal injury. All participants were informed about the study and gave their informed consent to participate. The participants’ flow diagram is presented in Figure 1.

### Randomization and blinding

Eligible women allocated according to a computer generated randomization list to one of the three groups: TTNS (Group 1); EMG-BF (Group 2); and a combined treatment group (Group 3). Allocations were sealed in opaque numbered envelopes. Randomization was performed using permuted blocks of 3 stratified by age. Due to the nature of the interventions, participants were not able to be blinded to the study interventions. However, the researchers who performed assessments of study outcomes and data analyses were blinded to the group assignments.

### Intervention

In the TTNS group (group 1), participants received bilateral transcutaneous stimulation of the posterior tibial nerve by the transcutaneous electrical nerve stimulation device (733A, Iran). Negative electrode was placed on the tibia, 2 cm superior from the medial malleolus. The positive electrode was placed in the middle of the medial foot arch. Treatment consisted of 15 sessions of electrical stimulation, 3 days a week, during a 5 weeks period. Stimulation was applied using biphasic square waves with a frequency of 10 Hz and a pulse width of 200 μs for 30 minutes. The current intensity increased up to the threshold of motor nerve stimulation, which was determined by the big toe flexion and below the patient’s discomfort threshold.

In the EMG-BF group (group 2), participants received BF using EMG-BF device (Neurotrack Mayo Plus 2, England) for 15 sessions, 3 days a week, during a 5 weeks period. The BF therapy performed in supine position and the probe placed inside the anal canal and targeted the external anal sphincter and Levator ani muscle (specifically the Puborectalis part). The aim of BF therapy was to educate the participants to perform appropriate and coordinate PFM contraction and relaxation using visual feedback and accomplish them with diaphragmatic breathing patterns. In this way, the initial five sessions was conducted by BF-guided education through teaching the participants exercising to tighten for 10 seconds and relax abdominal and pelvic muscles for 10 seconds and perform diaphragmatic breathing exercise, concurrently. In the last ten sessions, stronger and longer PFM contractions, better abdomino-pelvic muscle coordination, and completely relaxing the external anal sphincter provided using computer games with varying difficulty levels. The duration of treatment sessions was 20 min consisted of 40 cycles of contraction followed by relaxation. During mensuration, the participants were temporally withdraw from the session till the end of mensuration.

In Group 3, the combined treatment was provided identically to that outlined for Group1 and Group 2. Participants received 15 treatment sessions (3 day per week) over a period of 5 weeks including 30 min TTNS and 20 min EMG-BF.

### Outcome measures

Outcome assessments performed at baseline and immediately after 5, 10, and 15 treatment sessions. Outcome measures included a Persian version of the Wexner constipation scoring system questionnaire, 3-day bladder diary and EMG activity level (μv) of the pelvic floor muscle and external anal sphincter during maximal voluntary contraction (MVC), straining to defecate, and coughing.

The disease-specific Wexner constipation scoring system is reliable and valid questionnaire that measures chronic constipation severity^30^.

The 3-day bladder diary was used to measure the frequency of incontinence episodes during 3 consecutive days. The 3-day bladder diary showed good feasibility, reliability, and validity for assessing the lower urinary tract syndrome^31^.

EMG method using the anal probe was conducted to assess pelvic floor and sphincter muscle function in three conditions, including MVC, straining to defecate, and coughing. In MVC condition, participants were asked to maximally squeeze the anal probe in supine position while the vagina and anus were pulled upward without any surrounding muscles contraction. Maximal contraction was sustained for 5 seconds. In straining condition, the EMG activity was recorded while the participants were instructed to simulate defecation in the sitting position. In the coughing condition, participants were asked to perform coughing in a simulated manner in supine position. Each condition was performed in three trials with an interval of 30 seconds, and the average of three trials was recorded. The subjects were allowed to rest at least 1 min between conditions to prevent fatigue. Importantly, because the EMG amplitude influences by resting muscle tension, this resting value subtracted from the recorded signal during conditions.

### Statistical analysis

Statistical analyses were completed with SPSS (version 29.0). The normal distribution of dependent variables was confirmed by Kolmogorov-Smirnov test. One-way analysis of variance used to determine between group differences in demographic and clinical characteristics. A two-way repeated measure analysis of variance with one between (the three Groups) and one within (Time (baseline, session 5,10 and 15)) were used to determine if there were main or interaction effects of independent variables over treatment sessions. In addition, Tukey post hoc test was performed for the pairwise comparisons across the three groups and 4 times. A *P*-value <0.05 was considered statistically significant.

### Ethics approval and consent to participate

This study was approved by the Ethics Committee of Ahvaz Jundishapur University of Medical Sciences (Approval ID: IR.AJUMS.REC.1402.006) in accordance with the Declaration of Helsinki. All participants were informed about the study aims and procedures and provided written informed consent prior to participation.

## RESULTS

Eighty-seven women (both married and single) participated in the study. Baseline demographic and clinical characteristics were comparable across the three groups, with no statistically significant differences observed (Table 1).

**TABLE 1:** Demographic and Clinical Characteristics of the Study Participants.

Characteristics	TTNS group (n=29)	EMG-BF group (n=29)	Combined treatment group (n=29)	***P-*value**
Age, y	39.31	39.48	39.58	0.81
Height, cm	160.58	161.17	161.89	0.35
Weight, kg	67.44	67.75	71.89	0.11
No delivery	8	10	10	0.52
Vaginal delivery	8	9	11	0.66
Cesarean delivery	11	8	7	0.58
Vaginal & cesarean delivery	2	2	1	0.75
Number of children (More than two child)	9	13	11	0.45

TTNS: transcutaneous tibial nerve stimulation. BF: biofeedback.

Changes in constipation severity across the three groups over time are presented in Table 2.

**TABLE 2:** The Mean (SD) of Wexner Constipation Scoring System and 3-day bladder diary values.

Variable	Group	Mean ± SD				***P-*value between sessions**
		Baseline	Session 5	Session 10	Session 15	
**Wexner Constipation Scoring System**	TTNS	17.06±4.90	16.72±4.90	16.34±4.66	16.41±4.63	0.93
	**EMG-BF**	17.06±4.89	15.82±4.78	8.72±4.67	6.13±5.21	<0.001*
	**Combined treatment**	16.62±2.89	15.41±3.08	8.58±4.88	7.02±6.20	<0.001*
3-day bladder diary	TTNS	4.34±2.96	3.96±2.80	3.75±2.81	3.82±2.77	0.86
	**EMG-BF**	4.31±2.55	2.90±2.24	1.55±1.00	.43±.01	<0.001*
	Combined treatment	4.17±2.42	3.10±1.95	1. 6±1.29	.81±1.09	<0.001*

Significant P-values are marked with*. TTNS: transcutaneous tibial nerve stimulation. BF: biofeedback.

### Wexner constipation scoring system

The repeated measure analyses showed that the main effect of the group (F=7.39, *P*<0.001), the main effect of time (F=86.00, *P*<0.001), and the interaction of the group by time (F=17.41, *P*<0.001) were significant for the Wexner questionnaire (Table 3). Further analysis by ANOVA determined that the trend of changes during the treatment sessions was statistically significant for this variable in the EMG-BF (*P*<0.001) and combined treatment (*P*<0.001) groups (Table 3). Thus, the Tukey post hoc test indicated that the values of the Wexner questionnaire in the EMG-BF and combined treatment groups were significantly lower in session 10 (*P*<0.001) and session 15 (*P*<0.001) compared with their values before the treatment. Similarly, values of the Wexner questionnaire in the EMG-BF group were significantly lower in session 10 (*P*=0.011) and session 15 (*P*<0.001) compared with its value in session 5. Also, between-group analysis by the Tukey post hoc test showed significantly lower values of the Wexner questionnaire in the combined treatment group compared with TTNS (*P*<0.001) and in the EMG-BF group compared with TTNS (*P*=0.001) in session 10. Additionally, a lower value of the Wexner questionnaire was observed in the EMG-BF and combined treatment groups compared with the TTNS group in session 15 (*P*<0.001).

Table 3 presents the changes in the frequency of urinary incontinence episodes assessed by the 3-day bladder diary across treatment sessions.

**TABLE 3:** Summary of two-way repeated measure analysis of variance for Wexner Constipation Scoring System and 3-day bladder diary values.

Variable	The main effect of Group		The main effect of Time		Interaction of Group by time	
	F-ratio	***P*-value**	F-ratio	***P*-value**	F-ratio	***P*-value**
Wexner Constipation Scoring System	7.39	<0.001*	86.00	<0.001*	17.41	<0.001*
3-day bladder diary	3.90	<0.024*	72.99	<0.001*	9.99	<0.001*

Significant *P*-values are marked with*.

### Day Bladder Diary-3

The results showed that the main effect of the group (F=3.90, *P*=0.024), the main effect of time (F=72.99, *P*<0.001), and the interaction of the group by time (F=9.99, *P*<0.001) were significant for the 3-day bladder diary (TABLE 2). Further analysis by ANOVA determined that the trend of changes during the treatment sessions was statistically significant for this variable in the EMG-BF (*P*<0.001) and combined treatment (*P*<0.001) groups (TABLE 3). Thus, the Tukey post hoc test indicated that the values of the 3-day bladder diary in the EMG-BF and combined treatment groups were significantly lower in session 10 (*P*<0.001) and session 15 (*P*<0.001) compared with their values at baseline. Furthermore, between-group analysis by the Tukey post hoc test showed significantly lower values of 3DBD in the combined treatment group compared with TTNS (*P*=0.009) and in the EMG-BF group compared with the TTNS group (*P*=0.005) in session 10. Additionally, a lower value of the 3-day bladder diary in the EMG-BF and combined treatment groups compared with the TTNS group (*P*<0.001) has been found in session 15.

Group-by-time changes in pelvic floor EMG activity during maximum voluntary contraction (MVC) are summarized in Table 4.

**TABLE 4:** The Mean (SD) EMG-activity level.

Variable	Group	Mean ± SD				***P-*value between sessions**
		Baseline	Session 5	Session 10	Session 15	
EMG activity during maximum voluntary contraction	TTNS	14.32±5.62	14.41±5.72	14.10±5.3	13.88±5.17	0.98
	EMG-BF	13.89±6.49	19.96±8.40	26.08±11.58	34.05±14.07	<0.001*
	**Combined treatment**	12.47±8.42	18.38±8.91	24.50±10.28	31.79±14.16	<0.001*
EMG activity during straining	TTNS	8.55±3.60	8.57±3.57	8.54±3.59	8.27±3.27	0.98
	EMG-BF	8.41±4.37	7.01±2.99	5.35±2.71	3.70±2.48	<0.001*
	**Combined treatment**	7.4±3.63	6.36±2.72	4.84±2.09	3.93±2.18	<0.001*
EMG activity during coughing	TTNS	2.74±.91	2.89±.93	2.75±.81	2.69±.85	0.84
	**EMG-BF**	2.71±1.17	5.27±2.41	7.31±2.61	10.36±4.42	<0.001*
	**Combined treatment**	1.90±.91	4.00±1.74	8.25±3.71	11.94±6.36	<0.001*

Significant *P*-values are marked with*. TTNS: transcutaneous tibial nerve stimulation. BF: biofeedback.

### EMG activity during MVC

The repeated measure analyses of variance showed that the main effect of the group (F=9.85, *P*<0.001), the main effect of time (F=158.07, *P*<0.001), and the interaction of the group by time (F=42.64, *P*<0.001) were significant for the MVC (Table 5). Further analysis by ANOVA determined that the trend of changes during the treatment sessions was statistically significant for this variable in the EMG-BF (*P*<0.001) and the combined treatment (*P*<0.001) groups (Table 5). Consequently, the Tukey post hoc test indicated that the values of MVC in the EMG-BF group were significantly greater in session 10 (*P*<0.001) and session 15 (*P*<0.001) compared to its value before the treatment. Furthermore, the combined treatment group exhibited a significantly greater MVC in sessions 10 (*P*<0.001) and 15 (*P*<0.001) when compared to baseline value. Moreover, between-group analysis by the Tukey post hoc test showed significantly greater values of MVC in the EMG-BF group compared with TTNS in session 5 (*P*=0.025) and sessions 10 and 15 (*P*<0.001). In addition, greater values of MVC in the combined treatment group compared with TTNS group have been found in session 10 and 15 (*P*<0.001).

Table 5 illustrates changes in EMG activity during straining to defecate across the three groups.

**TABLE 5:** Summary of two-way repeated measure analysis of variance for EMG activity level: F-ratios and P-values by variable.

Variable	The main effect of Group		The main effect of Time			Interaction of Group by time	
	F-ratio	***P*-value**	F-ratio	***P*-value**	F-ratio	***P*-value**
EMG activity during Maximum voluntary contraction (μv)	9.85	<0.001*	158.07	<0.001*	42.64	<0.001*
EMG activity during straining(μv)	8.19	<0.001*	52.68	<0.001*	11.66	<0.001*
EMG activity during coughing(μv)	27.86	<0.001*	133.98	<0.001*	37.80	<0.001*

Significant *P*-values are marked with*

### EMG activity during straining

The repeated measure analyses showed that the main effect of the group (F=8.19, *P*<0.001), the main effect of time (F=52.68, *P*<0.001), and the interaction of the group by time (F=11.66, *P*<0.001) were significant for the activity level during straining (Table 4). Further analysis by ANOVA determined that the trend of changes during the treatment sessions was statistically significant for this variable in the EMG-BF (*P*<0.001) and combined treatment (*P*<0.001) groups (Table 5). Consequently, the Tukey post hoc test indicated that the values of activity level during straining in the EMG-BF group were significantly lower in session 10 (*P*=0.003) and session 15 (*P*<0.001) compared with its value before the treatment. Moreover, in the combined treatment group, activity level during straining was significantly lower in session 10 (*P*=0.02) and session 15 (*P*<0.001) compared with the pre-treatment baseline. Furthermore, in the combined treatment group, the activity level during straining was significantly lower in session 15 compared with session 5 (*P*=0.006). Also, between-group analysis by the Tukey post hoc test showed significantly lower values of activity level during straining in the combined treatment group compared with TTNS in session 5 (*P*=0.027), session 10 (*P*<0.001) and session 15 (*P*<0.001). Additionally, the EMG-BF group demonstrated significantly lower activity level during straining in session 10 (*P*<0.001) and session 15 (*P*<0.001) compared to the TTNS group.

Changes in pelvic floor EMG activity during coughing over the treatment period are presented in Table 5.

### EMG activity during coughing

The repeated measure analyses showed that the main effect of the group (F=27.86, *P*<0.001), the main effect of time (F=133.98, *P*<0.001), and the interaction of the group by time (F=37.80, *P*<0.001) were significant for the activity level during coughing (Table 4). Further analysis by ANOVA determined that the trend of changes during the treatment sessions was statistically significant for this variable in the EMG-BF (*P*<0.001) and combined treatment (*P*<0.001) groups (Table 5). Thus, the Tukey post hoc test indicated that the values of activity level during coughing in the EMG-BF group were significantly greater in session 5 (*P*=0.006), session 10 (*P*<0.001), and session 15 (*P*<0.001) compared with its value before the treatment. Additionally, in the combined treatment group, the activity level during coughing was significantly greater in session 10 (*P*<0.001) and session 15 (*P*<0.001) compared to the pre-treatment. Moreover, between-group analysis by the Tukey post hoc test showed significantly lower values of activity level during coughing in the combined treatment group compared with TTNS (*P*=0.007) and EMG-BF (*P*=0.010) groups before the treatment. Additionally, greater values of activity level during coughing in the EMG-BF group compared with TTNS (*P*<0.001) and combined treatment (*P*=0.025) groups have been found in session 5. Also, greater values of activity level during coughing in the EMG-BF group compared with TTNS (*P*<0.001) and in the combined treatment group compared with TTNS (*P*<0.001) have been found in sessions 10 and 15.

## DISCUSSION

This randomized controlled trial compared the effects of EMG-BF, TTNS, and their combination in women with coexisting dyssynergic defecation and stress urinary incontinence. The findings demonstrated that EMG-BF, either alone or combined with TTNS, resulted in significant improvements in constipation severity, urinary incontinence episodes, and pelvic floor muscle performance. In contrast, TTNS alone did not produce meaningful clinical or electromyographic improvements. These results underscore the central role of active pelvic floor muscle training and coordination in the management of concurrent bowel and urinary dysfunctions.

Accordingly, biofeedback therapy has been evaluated by several investigators. Chiarioni et al. in 2006 evaluated the efficacy of BF in comparison with continuous polyethylene glycol and concluded that BF is more effective and its benefits last at least 2 years^32^. In 2021, Özin et al. evaluated 24 DD patients according to Rome III criteria and concluded that anal canal pressure, BFT, colonic transit time, and quality of life significantly improved in biofeedback patients compared with controls^33^. Pulatova et al. in 2023 evaluated the effectiveness of PFM exercise plus biofeedback to PFM exercise alone in treating SUI in female patients in a systematic review study and concluded biofeedback addition to PRM exercise improved cure rate and PFM exercise strength without affecting leakage or QOL^34^. Wang et al. in 2024 evaluated 452 postpartum SUI women with new-onset and concluded that the efficacy of BF combined with PFM training was superior to PFM training alone^35^.

Overall, in current study positive effects of the BF therapy was significant after 10 treatment sessions (TABLE 2 AND 3). Women in the BF group learned how to relax their PFMs during straining to defecate and to increase their PFMs activity during coughing and other force full activity.

The key features of BF therapy are to increase awareness of the PFM and to help patients to learn to reliably contract and relax these muscles. As such, BF therapy aims to improve the function of the PFM strength, endurance, and coordination, thus providing better support to the pelvic organs. In DD, biofeedback has been efficacious for correction of paradoxical puborectalis and external anal sphincter contraction and restore normal defecation patterns^36^ ^,^ ^27^. In women with SUI, researchers reported the enhancement effect of biofeedback therapy on PFM training by improving the patients’ ability to selectively contract the pelvic floor, and consequently urethral closure pressure increasing and preventing urine leakage during stressful events^8^ ^,^ ^10^. Furthermore, the significant improvement in constipation severity and frequency of incontinence episodes supports the established role of BF therapy in alleviating constipation and urinary related symptoms, in concordance with the results reported by^37^ ^-^ ^41^.

The absence of significant benefit from TTNS on pelvic floor muscle activity and on defecatory and urinary symptoms represents another important finding of this study. TTNS primarily acts at a subconscious level^42^ and exerts its effects indirectly through neuromodulation of the sacral micturition and defecation reflex pathways via stimulation of the posterior tibial nerve, which projects to the sacral roots S2-S4 ^43^ ^,^ ^44^. In contrast, biofeedback therapy is a form of cognitive-behavioral intervention that requires patient motivation, attention, and active participation ^45^ ^,^ ^46^. This fundamental difference may explain the limited effectiveness of TTNS in improving coordinated pelvic floor muscle performance during straining, coughing, and voluntary contraction^47^ ^,^ ^48^ ^,^ ^49^ ^,^ ^50^ ^,^ ^51^ ^,^ ^52^.

However, no study has investigated the efficacy of TTNS specifically in SUI, and existing researches have focused exclusively on urgency-related urinary disorders. Interestingly, TTNS has been reported non-effective in older adults patients with functional UI resulted from mobility issues and severe cognitive impairment^52^.

These findings further highlight the positive efficacy of biofeedback therapy and suggest a negligible or absent additional role of TTNS in treatment planning for patients with coexisting dyssynergic defecation and stress urinary incontinence.

We acknowledge the following limitations. Because of cultural and religious restrictions, vaginal EMG-BF was precluded.

## CONCLUSION

EMG-BF, either alone or in combination with TTNS, significantly improved defecatory and urinary symptoms as well as pelvic floor muscle activity in women with coexisting dyssynergic defecation and stress urinary incontinence. The findings suggest that a minimum of ten treatment sessions is required to achieve clinically meaningful improvements. Active, skill-based pelvic floor muscle training appears to be the key determinant of therapeutic success in this population.

## Data Availability

Data-available-upon-request
